# Dynamical Behavior of Human α-Synuclein Studied by Quasielastic Neutron Scattering

**DOI:** 10.1371/journal.pone.0151447

**Published:** 2016-04-20

**Authors:** Satoru Fujiwara, Katsuya Araki, Tatsuhito Matsuo, Hisashi Yagi, Takeshi Yamada, Kaoru Shibata, Hideki Mochizuki

**Affiliations:** 1 Quantum Beam Science Center, Japan Atomic Energy Agency, Tokai, Ibaraki, Japan; 2 Department of Neurology, Osaka University Graduate School of Medicine, Suita, Osaka, Japan; 3 Center for Research on Green Sustainable Chemistry, Tottori University, Tottori, Japan; 4 Research Center for Neutron Science and Technology, CROSS-Tokai, Tokai, Ibaraki, Japan; 5 Neutron Science Section, J-PARC Center, Tokai, Ibaraki, Japan; University of Lincoln, UNITED KINGDOM

## Abstract

α-synuclein (αSyn) is a protein consisting of 140 amino acid residues and is abundant in the presynaptic nerve terminals in the brain. Although its precise function is unknown, the filamentous aggregates (amyloid fibrils) of αSyn have been shown to be involved in the pathogenesis of Parkinson's disease, which is a progressive neurodegenerative disorder. To understand the pathogenesis mechanism of this disease, the mechanism of the amyloid fibril formation of αSyn must be elucidated. Purified αSyn from bacterial expression is monomeric but intrinsically disordered in solution and forms amyloid fibrils under various conditions. As a first step toward elucidating the mechanism of the fibril formation of αSyn, we investigated dynamical behavior of the purified αSyn in the monomeric state and the fibril state using quasielastic neutron scattering (QENS). We prepared the solution sample of 9.5 mg/ml purified αSyn, and that of 46 mg/ml αSyn in the fibril state, both at pD 7.4 in D_2_O. The QENS experiments on these samples were performed using the near-backscattering spectrometer, BL02 (*DNA*), at the Materials and Life Science Facility at the Japan Accelerator Research Complex, Japan. Analysis of the QENS spectra obtained shows that diffusive global motions are observed in the monomeric state but largely suppressed in the fibril state. However, the amplitude of the side chain motion is shown to be larger in the fibril state than in the monomeric state. This implies that significant solvent space exists within the fibrils, which is attributed to the αSyn molecules within the fibrils having a distribution of conformations. The larger amplitude of the side chain motion in the fibril state than in the monomeric state implies that the fibril state is entropically favorable.

## Introduction

α-synuclein (αSyn) is a 140-amino acid protein, abundant in presynaptic terminals of nerve cells in the brain [1,2]. Although the exact function of this protein is unknown, it is implicated in the pathogenesis of Parkinson's disease (PD), which is a progressive neurodegenerative disorder [3]. Filamentous aggregates of αSyn are often found to be a major component of the protein deposits in the brain of patients with PD. These aggregates (amyloid fibrils) and/or the intermediate structures toward the mature fibrils of αSyn are thought to be related to the pathogenesis of PD. Moreover, αSyn is also associated with other neurodegenerative diseases such as dementia with Lewy bodies and multiple system atrophy. This diverse group of diseases is referred to as synucleinopathies. How αSyn can be toxic is a subject of intense studies. Elucidating the mechanism of the fibril formation of αSyn is thus important for understanding the mechanism of the pathogenesis of synucleinopathies, including PD.

When bacterially expressed *in vitro*, αSyn is intrinsically disordered in solution [4]. This recombinant protein forms amyloid fibrils, depending on the solution conditions under which the proteins are dispersed. Formation of amyloid fibrils in general involves partial unfolding of the proteins and the subsequent growth of the protofilaments and the mature fibrils, the latter possibly being formed by lateral association of the protofilaments [5]. The filamentous structures are stabilized by the stacked β sheets formed between the neighboring molecules (the cross-β structure) [6]. Involvement of the partial unfolding implies that the dynamics of the proteins plays an important role in the process of amyloid fibril formation. Proteins show a hierarchy of the dynamics, from local fluctuations of the side chains and loop motions at ps-to-ns time scales through domain motions at μs scales to conformational changes of proteins at ms scales [7]. Elucidating the role of the proteins dynamics requires a thorough understanding how the underlying fluctuations at the ps-to-ns scale are related to slower conformational changes. Inhibiting the dynamics at the ps-to-ns scale suppresses the activity of the protein [8]. Investigating the dynamics of these proteins during fibril formation is therefore important for elucidating the mechanism of this process. Amyloid fibril formation of αSyn involves the formation of a premolten globule-like partially folded intermediate, followed by the formation of the fibrils [9]. Elucidating possible variations in the dynamics of αSyn during the fibrillization process should provide insights into how the fibrils form, and the first step should be investigation of the dynamical properties of αSyn in the monomeric and fibril states. A recent study of αSyn in soluble and fibrillar forms using neutron scattering showed that different dynamic behavior of the soluble and fibrillar forms reflects its conformational heterogeneity [10].

Neutron scattering provides a unique tool to directly measure the dynamics of proteins at ps-to-ns time scales and ångstrom length scales [11]. Incoherent neutron scattering arises predominantly from hydrogen atoms because the scattering cross-section of hydrogen atoms is ~40 times larger than that of other atoms. Incoherent quasielastic neutron scattering (QENS) provides information on the correlation times and the amplitudes of diffusive motions of atoms. Because about half of the atoms in the proteins are hydrogen atoms and because they are pseudo-homogeneously distributed in the protein, QENS provides information on the average motion of (the hydrogen atoms within) the whole protein. Many studies have been conducted using the QENS approach to obtain insights into how the protein dynamics at ps-to-ns time scales is related to functionally relevant structural changes, including, for example, studies related to protein folding [12–20], the photosynthesis systems [21–23], hemoglobin [24], and bacteriorhodopsin during its photocycle [25]. Furthermore, the studies combining QENS with other techniques such as NMR [26] and molecular dynamics simulation [21,27] show that the consistent results can be obtained with these techniques. These studies demonstrate usefulness of QENS as well as other incoherent techniques of neutron scattering including elastic incoherent neutron scattering and inelastic neutron scattering [11,28].

In the present study, we made a comparison between the dynamical properties of αSyn in the monomeric and fibril states. For this purpose, we carried out the QENS experiments of the solution samples of αSyn in the monomeric and fibril states under the similar experimental conditions. Analysis of the QENS spectra obtained reveals differences in the dynamics of αSyn between these states.

## Materials and Methods

### Preparation of the samples

Human wild-type αSyn was expressed in *E*. *coli* BL21(DE3), and purified as described [29]. The purified protein was lyophilized, and stored at −80°C. The stored protein was dissolved with the PBS buffer (10 mM phosphate buffer (pD 7.4), 137 mM NaCl, and 2.7 mM KCl) in D_2_O at the concentration of about 15 mg/ml. The solution was ultracentrifuged at 100,000 × g for 20 min to remove any aggregates just before starting the neutron scattering measurements. This solution sample is thus considered to be monomeric. The final concentration of this solution was 9.5 mg/ml. The concentration of αSyn was determined spectrophotometrically using an extinction coefficient of ε_280 nm_^0.1%^ = 0.354 [30]. About 2 ml of this sample solution was put into a double-cylindrical aluminum cell with the sample thickness of 1.0 mm, and sealed with indium wire.

Amyloid fibrils of αSyn were prepared from the αSyn solution in the PBS buffer as described [31]. The solution containing the fibrils of αSyn was centrifuged at 10,000 × g for 10 min. The pellets obtained were then put into a well with a size of 29 mm × 38 mm of a flat aluminum cell, and sealed with an aluminum lid and indium wire. The thickness of the sample in this cell was 0.5 mm. The concentration of αSyn in the pellets was determined to be 46 mg/ml from the volume and the protein concentration of the solution before the formation of fibrils and those of the supernatant after centrifugation.

Characterization of αSyn in the monomeric state and fibril states was done by circular dichroism (CD) measurements, dynamic light scattering (DLS) measurements on the solution of αSyn in the monomeric state, and transmission electron microscopy (TEM) imaging of the αSyn fibrils. For the CD measurements, the solutions in both states were diluted to about 0.3 mg/ml, and the CD spectra of these diluted solutions were acquired using a JASCO J-820 spectropolarimeter (JASCO, Tokyo, Japan). The measurements were carried out at 25°C, using a quartz cuvette with a path length of 1 mm. The DLS measurements were carried out on a solution of αSyn under the condition similar to that employed for the QENS measurements (9.0 mg/ml of αSyn in the PBS buffer in D_2_O), using a system consisting of a 22 mW He-Ne laser (wavelength, λ = 632.8 nm), an avalanche photodiode mounted on a static/dynamic compact goniometer, ALV/LSE-5003 electronics and an ALV-5000 correlator (ALV, Langen, Germany). The measurements were made at scattering angles from 30° to 120° in 15° steps, at temperatures at 15°C, 20°C, and 25°C. The CONTIN analysis [32] was employed for data analysis. The TEM images of αSyn in the fibril state were acquired using a JEM-1400Plus (JEOL, Tokyo, Japan) TEM operated at 80 kV. 5 μl of the sample solution was placed on a copper grid (400-mesh) covered by a carbon-coated collodion film (Nisshin EM, Tokyo, Japan) for 60 s. Excess sample solution was removed by blotting with filter-paper and air drying. The results of these measurements are shown in S1 Fig.

As shown in S1(a) Fig, the CD spectrum of αSyn in the monomeric state has characteristics typical of intrinsically disordered proteins. Moreover, the number distribution of the hydrodynamic radius of αSyn in the monomeric state (S1(c) Fig) reveals a single-peak distribution with a small tail extending toward larger radii, indicating that most of the proteins are in the monomeric state with a small fraction of (possible) oligomers. These results indicate that in the monomeric state, most αSyn molecules are indeed monomeric. Note, however, that αSyn in this state contains about 10% fraction of the oligomers (see the caption of S1 Fig). On the other hand, the CD spectrum of αSyn in the fibril state is characteristic of β-sheet structures (S1(b) Fig), and the TEM image (S1(d) Fig) clearly shows the existence of fibrils. These results indicate that in the fibril state, αSyn molecules indeed form fibrils, the structures of which are stabilized by the cross-β structures.

### Quasielastic neutron scattering experiments

QENS measurements were carried out using the near-backscattering spectrometer *DNA* at beam line BL02 [33] at the Materials and Life Science Experimental Facility at the Japan Proton Accelerator Research Complex (MLF/J-PARC), Tokai, Ibaraki, Japan, run at 300 kW. The spectra over an energy transfer range from −0.5 meV to 1.5 meV were measured at an energy resolution of 12 μeV. This energy resolution corresponds to a time window of ~55 ps, which means that the motions faster than ~55 ps are accessible with this setup. The measurements were carried out on a solution of αSyn in the PBS buffer in the cylindrical cell, pellets of αSyn in the fibril state in the flat cell, the PBS buffer in the cylindrical cell, and the PBS buffer in the flat cell, at several temperature points between 280 K and 300 K. The size of the incident neutron beam was 20 mm × 20 mm. The exposure time was about 6 hours for a single measurement. The spectra of the empty cell were subtracted from the spectra of the samples and the buffers, with the transmission values as the scaling factors. The spectra obtained were then normalized to those of the vanadium standard. The spectra of the PBS buffer in D_2_O were then subtracted from those of the samples with appropriate scaling factors. The scaling factors were determined from the total neutron scattering cross-section of the samples, calculated from the chemical composition of αSyn, the concentrations of αSyn in the samples, and the chemical compositions and the concentrations of the buffers (including D_2_O). Details of how the scaling factors were estimated are described in S1 Text. The data reduction was done separately for the data of the samples in the cylindrical cells and those in the flat cells. The data reduction was carried out using the software "Utsusemi", developed for the data treatment at MLF/J-PARC [34]. The available range of the momentum transfer Q (= 4πsinθ∕λ, where 2θ is the scattering angle, and λ is the wavelength of the incident neutrons) from these measurements was between 0.125 Å^-1^ and 1.775 Å^-1^. In spectra in the region Q < ~0.45 Å^-1^, however, the contribution from coherent scattering is not negligible [35,36]. In addition, the spectra in the region Q > 1.4 Å^-1^ had insufficient statistics to be properly analyzed. Analysis of the spectra in the Q-region between 0.45 Å^-1^ and 1.4 Å^-1^ is thus described here. Note that we examined various possible systematic errors that could distort the resulting spectra, and verified that these possible errors were negligible (see S2 Text).

## Results

**Fig 1:**
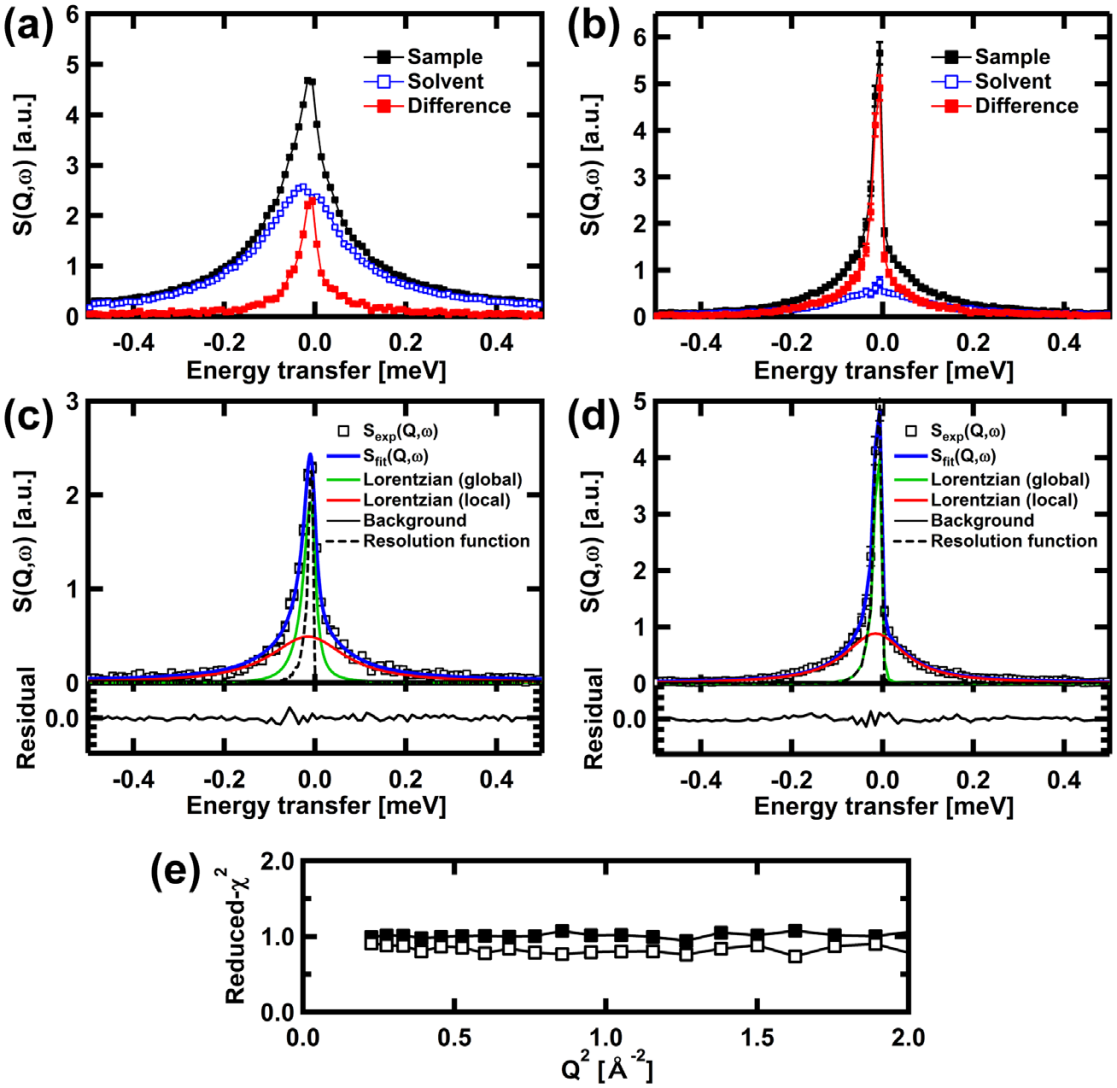
Examples of quasielastic neutron scattering spectra, S(Q,ω). The spectra of the samples, the solvent, and the difference spectra between the sample and the solvent, of αSyn in (a) the monomeric state and (b) the fibril state, at Q = 1.225 Å^-1^ and at 280 K, are shown. Filled symbols in black and open symbols in blue show the spectra of the sample solutions and those of the solvent, respectively. The spectra of the empty cell were subtracted from these spectra. Filled symbols in red show the difference spectra between the sample and the solvent. (c) Fits using Eq 1 to the difference spectra, which are due to monomeric αSyn molecules in the sample solution. (d) Fits to the difference spectra, which are due to αSyn molecules in the fibril state. In the upper panels, open squares denote the data, thick solid lines in blue denote the total fits, solid lines in green and red denote the narrow and wide Lorentzian functions, corresponding to *L*_global_(Q,ω) and *L*_local_(Q,ω), respectively, thin solid lines in blue show the background, and dashed lines in black show the resolution functions. The lower panels show the residuals of the fits. (e) Reduced-χ^2^ of the fits to the data. Filled squares and open squares are for the values to the data of αSyn in the monomeric state and the fibril state, respectively, at 280 K. Note that similar values of Reduced-χ^2^ were obtained for the data measured at other temperatures.

Fig 1(a) and 1(b) shows examples of QENS spectra from αSyn in the monomeric state and in the fibril state, respectively. The spectra of the sample solution, the solvent, and the difference spectra between them at Q = 1.225 Å^-1^ at 280 K are shown. The difference spectra, which correspond to the spectra of the αSyn molecules, are well above the noise level. The spectra, S(Q,ω), at each Q value are fit with the equation [37],

$$\text{S}\left(\text{Q},\omega \right)=\left[A_{0}\left(\text{Q}\right)\delta \left(\omega \right)+\left\{1-A_{0}\left(\text{Q}\right)\right\}L_{\text{local}}\left(\text{Q},\omega \right)\right]⊗L_{\text{global}}\left(\text{Q},\omega \right)⊗RF\left(\text{Q}\right)+BG,$$ (1)

where *A*_0_(Q) is the elastic incoherent structure factor, which characterizes the geometry of the motions observed, δ(ω) is the Dirac delta function designating the elastic peak, *L*_local_(Q,ω) and *L*_global_(Q,ω) are the Lorentzian functions ((1/π)*(Γ/(ω^2^+Γ^2^)), where Γ is the half width at half maximum (HWHM), which is Q-dependent), characterizing the local internal motion and the global diffusive motion of the protein, respectively, ⊗ denotes the convolution operation, *RF*(Q) is the resolution function obtained from the vanadium measurements, and *BG* is the background. The results of the fits are shown in Fig 1(c) and 1(d). The residuals of the fits shown in the lower panels of Fig 1(c) and 1(d) testify to the quality of the fit, as does the low values of the reduced-χ^2^ ~1.0 shown in Fig 1(e).

**Fig 2:**
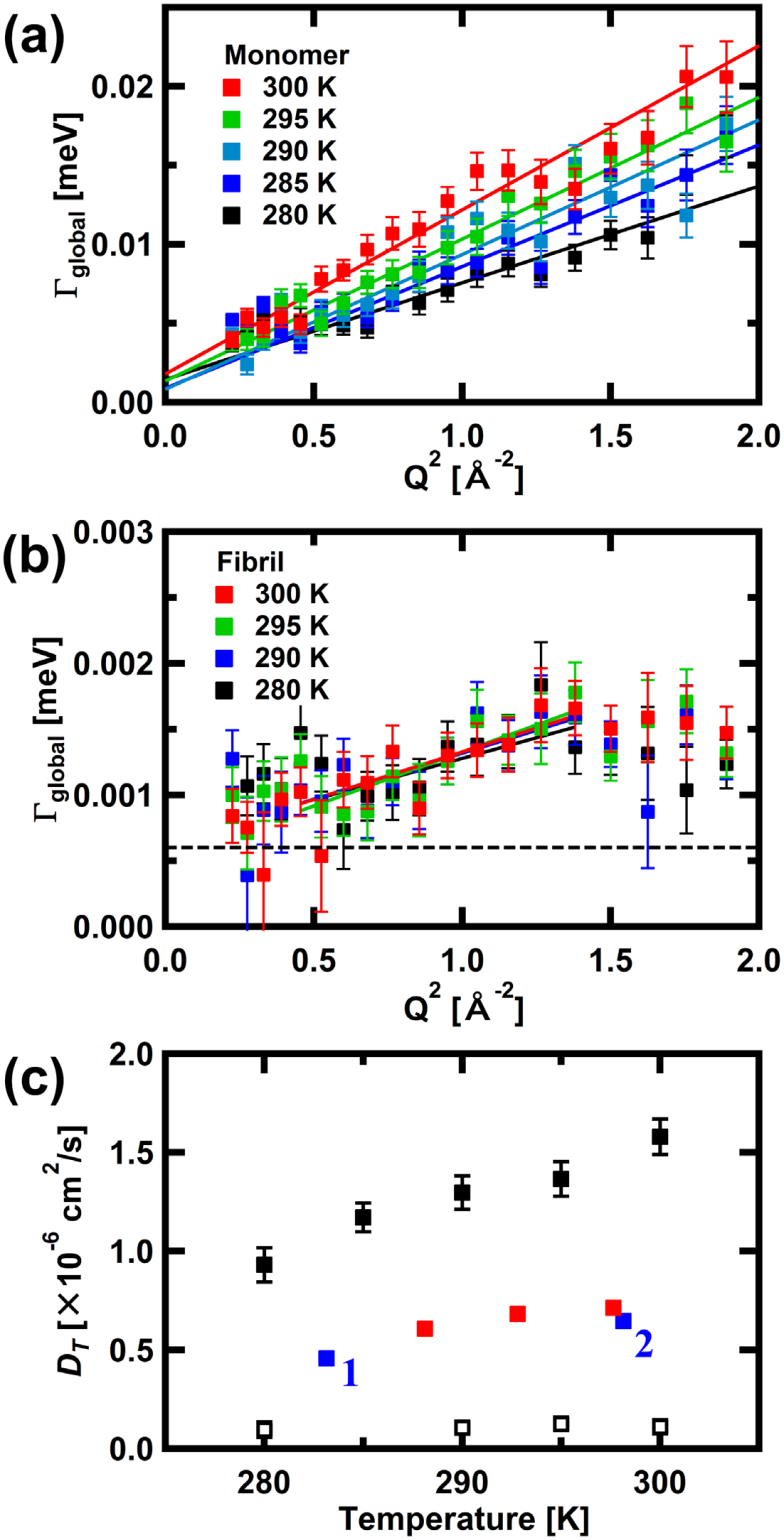
Variations in the HWHM, Γ_global_, of *L*_global_(Q,ω) as a function of Q^2^ for αSyn in (a) the monomeric state and (b) the fibril state. Solid lines are the linear fits to the data, the slopes of which provide estimates of the effective diffusion coefficients, *D*_*T*_. The dashed line in (b) is 1/10 × HWHM for the resolution function. The error bars are within in symbols where not shown. (c) Summary of *D*_*T*_ for αSyn. Filled and open squares in black are for the monomeric state and the fibril state, respectively. The filled squares in blue with subscripts 1 and 2 are the *D*_*T*_ values estimated for αSyn in solution, reported in Refs. 40 and 41, respectively. Note that these values were corrected for the differences in solvent viscosity due to the different D_2_O concentration in the samples for the NMR measurements (6% and 10% D_2_O for Refs. 40 and 41, respectively) and the QENS measurements (100% D_2_O) in this study. Filled squares in red denote the *D*_*T*_ values estimated from the DLS measurements on the αSyn solution under the condition similar to that employed for the QENS measurements (9.0 mg/ml αSyn in the PBS buffer in D_2_O) done in this study.

As shown in Fig 1(c), significant broadening of the elastic peak by *L*_global_(Q,ω) in the spectra of αSyn in the monomeric state is observed. On the other hand, such broadening is not significant in the fibril state. (Note that the examples of the QENS spectra along with the fits using Eq 1 at all the temperatures measured are summarized in S2 Fig) Fig 2(a) shows the HWHM, Γ_global_, of *L*_global_(Q,ω) of αSyn in the monomeric state. The Q^2^-dependence of Γ_global_ can be fit by a linear relationship, Γ_global_ = *D*_*T*_Q^2^, where *D*_*T*_ denotes the effective diffusion coefficient. This relationship is expected if the motion described by *L*_global_(Q,ω) is free diffusion. The values of *D*_*T*_ obtained are shown in Fig 2(c). Fig 2(b) shows Γ_global_ for αSyn in the fibril state. Γ_global_ is constant in the low-Q^2^ region, and increases asymptotically to a plateau at high Q^2^. This behavior is characteristic of diffusion in a confined space [37]. The linear fits to the data between Q^2^ = 0.4 Å^-2^ and 1.4 Å^-2^ provided the *D*_*T*_ values in the fibril state. These values are also shown in Fig 2(c). Note that the Γ_global_ values in the fibril state were about one-sixth of the HWHM of the resolution function. Considering, however, the fact that broadening by Lorentzian for about one tenth of the resolution function can be detected [38,39], these Γ_global_ values appear to reflect the diffusive motion of the αSyn molecules in the fibrils.

The *D*_*T*_ values of αSyn in the monomeric state are around 1–1.6 × 10^−6^ cm^2^/s. On the other hand, the published values of *D*_*T*_ estimated from the NMR measurements [40,41] are smaller than the values obtained here. We also estimated *D*_*T*_ from the DLS measurements of αSyn solution under the condition similar to that employed for the QENS measurements. The published values of *D*_*T*_ and those obtained from our DLS measurements are also shown in Fig 2(c). The values obtained from the DLS measurements are consistent with the published values from NMR. The *D*_*T*_ values from the QENS measurements and those from NMR and DLS thus differ by a factor of about two. Whereas *D*_*T*_ obtained from NMR and DLS is the translational diffusion coefficient, *D*_*T*_ obtained from QENS measurements contains contributions from rotational motion. It was shown analytically that the *D*_*T*_ values of hard spheres from QENS are about 1.27 times larger than the translational diffusion coefficients due to the contribution of rotational diffusion [38]. The contribution of rotational diffusion to the diffusion coefficient is also observed in QENS measurements of the globular protein, bovine serum albumin [42]. For anisotropic particles, an increase in anisotropy increases the hydrodynamic radius and thereby decreases the rotational diffusion coefficient. This situation for rigid particles, however, may not be applicable to flexible molecules. Intrinsically disordered proteins such as αSyn undergo various types of motions, including segmental motion and long-range correlated motion, in addition to the local side-chain motion and overall diffusive motion, and these different types of motions contribute to the overall rotational correlation time [43]. Such complicated behavior could be the reason why the difference between *D*_*T*_ and the translational diffusion coefficient of αSyn is larger than the globular proteins. A recent study of the intrinsically disordered protein, myelin basic protein (MBP), using neutron spin-echo spectroscopy [44] showed that the internal dynamics such as stretching and bending motions of the polypeptide backbone contributes significantly to the overall diffusion. The observed diffusion coefficient of MBP is indeed about 1.7 times larger than the translational diffusion alone, which is consistent with our observations.

In contrast, the *D*_*T*_ values in the fibril state are in the order of 10^−8^ cm^2^/s. This indicates that the global diffusive motion observed for αSyn molecules in the monomeric state is largely suppressed, leaving only slow diffusive motion in a confined volume.

**Fig 3:**
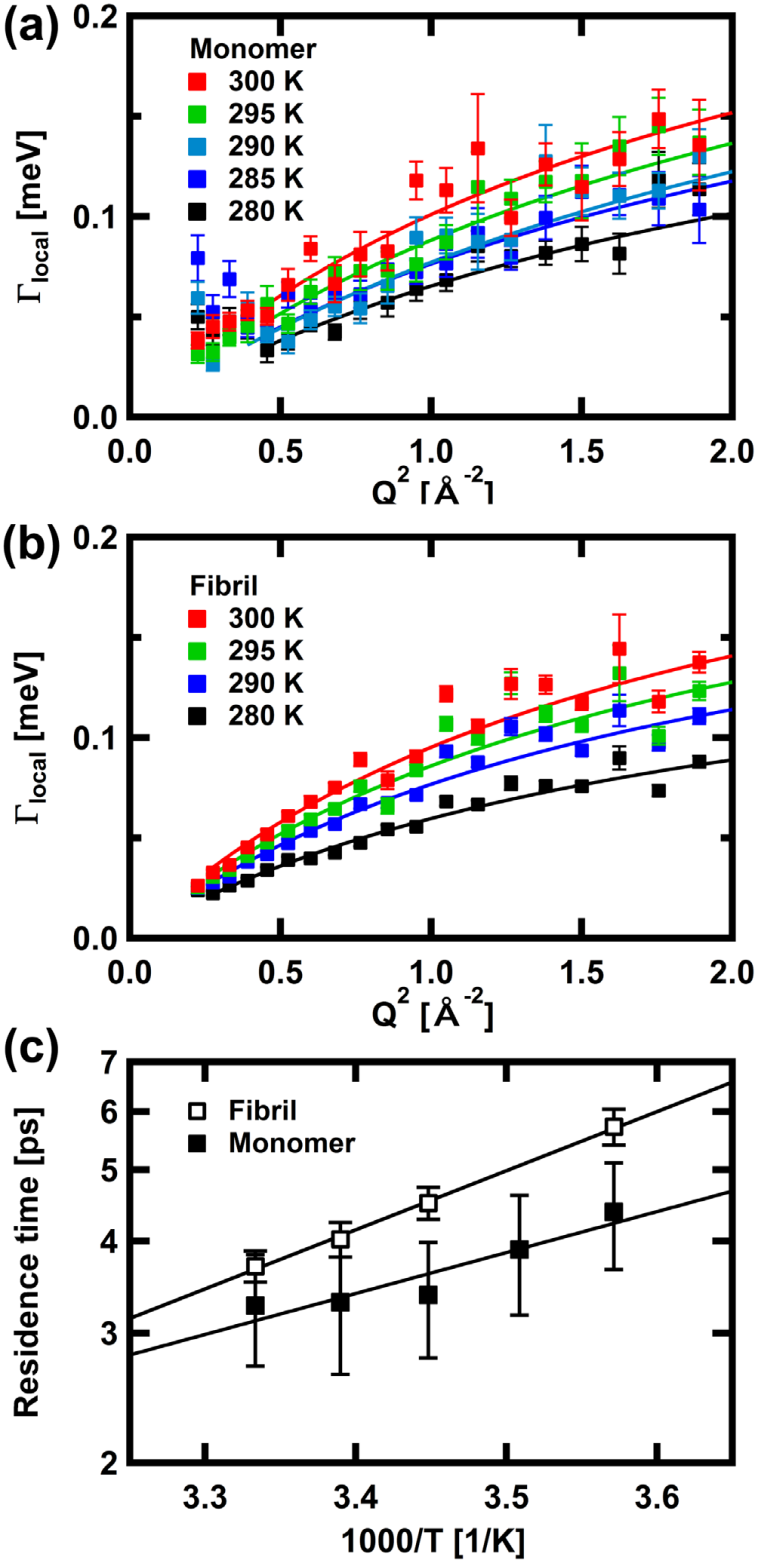
The Q^2^ dependences of the HWHM values, Γ_local_, of the wide Lorentzian functions of αSyn in (a) the monomeric state and (b) the fibril state. Solid lines are the fits with the equation based on a jump-diffusion model. (c) Arrhenius plots of the residence time of the jump diffusion model, estimated from the fits shown in (a) and (b). In (a), (b), and (c), the error bars are within in symbols where not shown.

Whereas *L*_global_(Q,ω) represents the motions of the whole protein and the backbone motions such as the segmental motions, another Lorentzian function, *L*_local_(Q,ω), represents faster motion than that represented by *L*_global_(Q,ω), such as side-chain fluctuations. Fig 3(a) and 3(b) shows Γ_local_, the HWHM of *L*_local_(Q,ω), as a function of Q^2^. Γ_local_ of αSyn in the monomeric state appears to be constant in the low-Q^2^ region, and approach asymptotically a plateau at high Q^2^ though it does not reach the plateau in the observed Q^2^-range. Γ_local_ of αSyn in the fibril state exhibits similar behavior though the constant region in the low-Q^2^ region is not clear. The constant region is likely to occur for Q less than the minimum Q observed. This low-Q behavior implies the motion described by *L*_local_(Q,ω) is diffusive motion within a confined space [37]. The size of the confined space is given by a relationship, Q_max_ = π/*a*_0,_ where Q_max_ is the maximum value of Q for which Γ_local_ is constant, and *a*_0_ is the radius of the confined sphere, assuming a spherical confined space [45]. The Q_max_ value for αSyn in the monomeric state is about 0.65 Å^-1^, whereas that for αSyn in the fibril state is less than 0.47 Å^-1^. These values correspond to the radius of 4.8 Å and 6.7 Å, respectively. The confined space in which local diffusive motion is possible is thus larger in the fibril state than in the monomeric state.

The asymptotic approach of Γ_local_ to a plateau at high Q implies that the jumping nature of the side chain motion becomes evident with increasing Q. This behavior is approximated by the equation based on the jump-diffusion model, Γ = *D*Q^2^/(1+*D*Q^2^τ), where *D* is the jump-diffusion coefficient and τ is the residence time [37]. The residence time is estimated from fits to the data for Q^2^ between 0.5 Å^-2^ and 2.0 Å^-2^ for αSyn in the monomeric state and between 0.25 Å^-2^ and 2.0 Å^-2^, for αSyn in the fibril state. The residence time is related to the height of the potential barrier of the jumps through the Arrhenius law, τ = τ_0_exp(-*E*_a_/*k*_*B*_*T*), where *E*_a_ is the activation energy, which is a measure of the mean height of the potential barrier, and *k*_*B*_ is the Boltzmann constant. Fig 3(c) shows Arrhenius plots of the residence time. Larger residence times in the fibril state indicate that the local motion is slower in the fibril state than that in the monomeric state. The activation energy is estimated to be 2.6 ± 1.9 kcal/mol and 3.7 ± 0.7 kcal/mol for the monomeric and the fibril states, respectively, suggesting that the jumping process was similar between the monomeric state and the fibril state.

**Fig 4:**
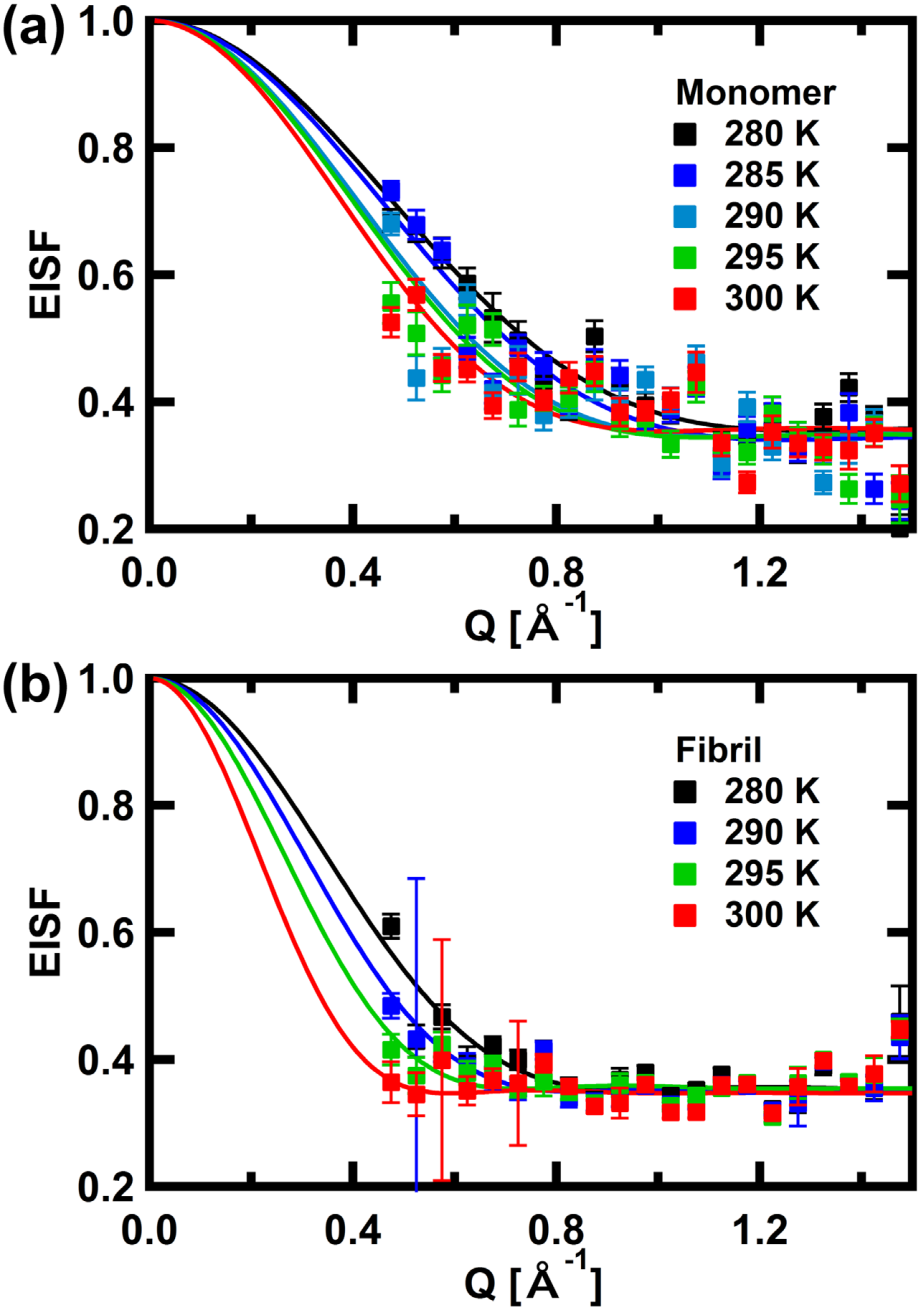
The EISF curves of αSyn in (a) the monomeric state and (b) the fibril state. Solid lines are the fits with Eq 2. The error bars are within in symbols where not shown.

The geometry of these local motions can be characterized from the elastic incoherent structure factor (EISF, *A*_0_(Q) in Eq 1). For each Q, *A*_0_(Q) can be calculated as the ratio of the intensity of the elastic peak to the sum of the intensity of the elastic peak and that of the quasielastic scattering. Plots of the EISF against Q are shown in Fig 4. These curves are fit with the equation based on the diffusion in a confined sphere model [45],

$$\text{EISF}\left(\text{Q}\right)=p+\left(1-p\right)\times {\left(3j_{1}\left(\text{Q}a\right)/\text{Q}a\right)}^{2},$$ (2)

where *p* is the fraction of "frozen" atoms, which appear to be immobile because the frequency of the motion is outside of the accessible time scale employed here, *j*_1_(Q*a*) is the first-order spherical Bessel function of the first kind, and *a* denotes the radius of the confined sphere.

**Fig 5:**
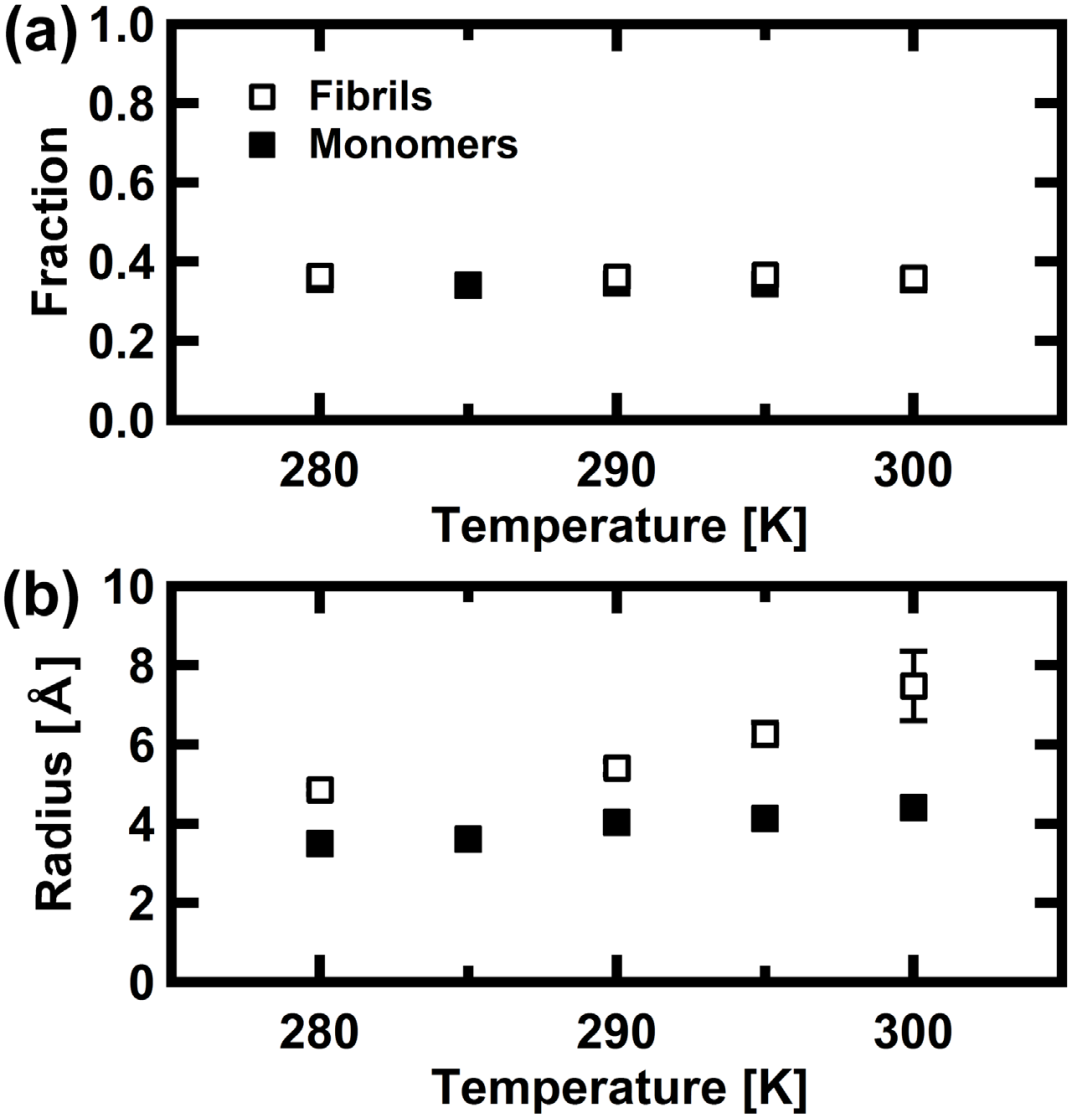
Summary of parameters estimated from the fits to the EISF curves. The parameters calculated using Eq 2, shown in Fig 4 are shown. The parameters shown are (a) the fraction of frozen atoms (*p* in Eq 2), and (b) the radius of the confined sphere (*a* in Eq 2).

The parameters obtained from these fits are summarized in Fig 5. The fraction of frozen atoms in the monomeric state is similar to that in the fibril states. On the other hand, the radius of the confined sphere is significantly larger in the fibril state than in the monomeric state, which indicates that the amplitude of the local motion is significantly larger in the fibril state. These values are comparable to those estimated from the Q^2^-dependences of Γ_local_ shown in Fig 3(a) and 3(b). Thus, analyses of the widths of the Lorentzians and the EISF give consistent results.

## Discussion

We carried out the QENS measurements on the αSyn solutions with concentrations of about 10 mg/ml and 46 mg/ml for αSyn in the monomeric state and the fibril state, respectively. Feasibility of the QENS measurements on dilute protein solutions should greatly expand the possibility of QENS studies in biochemical context. As shown in S1 Fig, most of the αSyn molecules were monomeric in the monomeric state, whereas the molecules in the fibril state indeed formed fibrils. Since the fibril-state sample was prepared by centrifugation and took the form of pellets, it is unlikely that it contained a significant fraction of the molecules in intermediate states. Note that the sample in the monomeric state contains about 10% fraction of the oligomers. Since QENS observes the average dynamics, the results for αSyn in the monomeric state contain the contributions from the oligomers. In case that the oligomers have similar dynamical properties to those of the monomers, the obtained results reflect the properties of the monomers. On the other hand, if the oligomers have similar properties to those of the fibrils, the results are biased toward the properties of the fibrils. In this case, the differences between the monomers and the fibrils could be more significant than those observed in this study. The differences detected by the QENS measurements here should thus reflect the differences between the monomeric and fibril states.

The QENS spectra were analyzed using the phenomenological equation containing two Lorentzian functions, *L*_global_(Q,ω) and *L*_local_(Q,ω), which represent the global diffusive motions of the entire molecules and local dynamics within the molecules such as the side chain motions, respectively. Each Lorentzian function stands as an average of the distribution of the complex motions that the atoms within the protein undergo. As referred to as the time window above, the accessible motions with the QENS measurements are restricted to those faster than the time specified by the instrumental energy resolution and those slower than the motions, whose corresponding Lorentzian functions have too wide the widths to be detected. The averaged views of the dynamics provided by the QENS measurements thus depend on this time window. Nevertheless, the information obtained is valid for investigating the changes in the dynamics between the different structural states.

For the global motions in the monomeric state, not only the translational diffusion of the entire molecules but also the complicated behavior due to the segmental motions and long-range correlated motions within the molecules are suggested to contribute to the narrow Lorentzian function, *L*_global_(Q,ω). Contributions of these various motions result in the larger diffusion coefficient than the translational diffusion coefficient obtained by other techniques. Formation of the fibrils makes these global motions very slow so that quasielstic broadening due to these motions goes within the instrumental resolution function. Only part of these global motions that are fast enough to leak out of the resolution function were observed.

The local motions, on the other hand, show a different trend. The HWHM of *L*_local_(Q,ω) indicates that the diffusive motion within a confined space occurs in both the monomeric and fibril states. The local side chain motions are convoluted with the slower motions of the regions in the backbone, to which the side chains are bound. Since whether each motion contributes to *L*_local_(Q,ω) or *L*_global_(Q,ω) depends on the relaxation time of the motion, the slower motion of the backbone can contribute mainly to the global motion, whereas the side chain motions contribute mainly to the local motion. The local motion can thus be regarded as the motion of the side chains bound to a "fixed" backbone, and the amplitude of such local motion can be confined.

Although the residence time of the local motion is somewhat longer in the fibril state, the activation energy of this motion estimated from the Arrhenius plots is similar between the monomeric and fibril states. This energy is comparable to that for water molecules [46], suggesting that the local motion involves hydrogen bond fluctuations, as observed in hemoglobin [47]. This observation implies that the side chains of αSyn are surrounded by water molecules even in the fibril state. The dynamics of intrinsically disordered proteins, such as the tau protein and the casein protein, has been explored using neutron scattering, and the factors that affect the dynamics have been investigated [39,48,49]. In particular, the dynamics of hydration water was found to couple with the dynamics of the intrinsically disordered proteins [48]. Such coupling between the hydration water dynamics and the protein dynamics is likely to remain in the fibril state. It should be noted that even the cross-β structures in the cores in αSyn fibrils contains water molecules whereas those of other proteins are completely dry [50], suggesting the importance of water molecules in the fibril formation.

Both the Q-dependence of the width of the Lorentzian and the EISF curves consistently indicate that the amplitude of the local motion is larger in the fibril state than in the monomeric state. Such large amplitude of the local motion requires a solvent-filled space within the fibrils large enough to accommodate such motion. The fact that the N- and C-terminal regions in αSyn remain disordered and flexible in the fibril state [51,52] indicated that αSyn molecules adopt various conformations in the fibrils. The fibrillar structures, in which molecules with a distribution of conformations are held by the cross-β structure [53], should thus contain large spaces between the molecules within the fibrils. A recent neutron scattering study of the fibrils of the intrinsically disordered tau protein showed that hydration water dynamics is enhanced in the fibrils [54]. Considering the coupling of the water dynamics and the protein dynamics, this observation is consistent with our results, which reflect the larger amplitude motion in the fibril state than in the monomeric state. Although they did not detect enhanced protein dynamics in the fibrils, this is not necessarily a contradiction: the different experimental conditions can explain this apparent discrepancy. They measured the mean square displacement, which contains contributions from the backbone atoms as well as the side chain atoms, in the hydrated powder sample whereas we observed the local side chain motion in the (pelleted) solution sample.

The radius *a* of the confined sphere represents the amplitude of the local motion. This parameter can be used to estimate the conformational entropy: in particular, the entropy change, Δ*S*_Conf_, due to the conformational change from the conformation 1 to the conformation 2 can be estimated by the equation [14],

$$\text{Δ}S_{\text{Conf}}=3R\text{ln}\left(a_{2}/a_{1}\right),$$ (3)

where *R* is the gas constant, and *a*_1_ and *a*_2_ are the radii of the confined spheres of conformation 1 and conformation 2, respectively. Using the *a* values of the monomeric and fibril states shown in Fig 5 for *a*_1_ and *a*_2_ in Eq 3, respectively, the entropy change due to the fibril formation is estimated to be 8.5 ± 0.9 JK^-1^mol^-1^ per residue, 7.1 ± 1.0 JK^-1^mol^-1^ per residue, 10.1 ± 1.3 JK^-1^mol^-1^ per residue, and 13.6 ± 3.2 JK^-1^mol^-1^ per residue, at 280 K, 290 K, 295 K and 300 K, respectively. The *a* values used here were obtained for the side chain motion accessible within the time window in the experimental setup employed here. Contributions from frozen atoms were not included. Frozen atoms include atoms, whose motion contributes to the narrow Lorentzian function. This broadening arises from the diffusion of the entire molecule as well as the segmental motion and the long-range correlated motion of the backbone. The frozen atoms should thus include the atoms in the backbone and those in the side chains, whose motion is coupled to the motion of the backbone.

The backbone entropy change cannot be estimated from the measurements carried out here because the parameter corresponding to *a* for the backbone motion is not available from these measurements. A recent estimate of the backbone entropy change by protein folding [55] shows that the entropy loss by folding is 14–15 JK^-1^mol^-1^ per residue at 300 K. It appears that the side-chain entropy gain by fibril formation (13.6 ± 3.2 JK^-1^mol^-1^ per residue at 300 K) is counterbalanced by the backbone entropy loss of folding (note that here the monomeric state corresponds to the unfolded state and the fibril state corresponds to the folded state.). Hydrogen-deuterium exchange studies, however, show that in the fibril state, only a middle segment (the residues 39–101) of αSyn is protected whereas in the monomeric state, no protected regions are detected [52,56]. This result indicates that folding occurs only in this middle region by fibril formation and other N- and C-terminal regions remain flexible. This was also shown by solid-state NMR [51,52]. Thus, in the fibril state, about 45% of the residues of αSyn are folded. The change in the backbone entropy due to fibril formation of αSyn is therefore 6.3–6.8 JK^-1^mol^-1^ per residue at 300 K. Note that this value should be a maximum value because αSyn in the monomeric state is not completely unfolded [9].

From the chemical composition of αSyn (see S1 Text), the number of H in the backbone (including Gly) is counted to be 158, which is 20% of the total number of H in αSyn. The fraction of frozen atoms is, on the other hand, about 35% in both the monomeric and fibril states as shown in Fig 5(a). The remaining 15%, which corresponds to about 118 H atoms, comes from the side chains. Since the average number of H contained in each side chain in αSyn is calculated to be 5.1, the side chains of about 23 residues contribute to frozen atoms. (Note that these estimates are virtually the same if all atoms are used in the estimation.) It is impossible to calculate the side-chain entropy change of these residues. We, however, assume here that these side chains contribute to the entropy loss by fibril formation, because combining this assumed loss and the backbone entropy loss estimated above, which is a maximum, with the side-chain entropy gain estimated above provides a minimum estimate of the total entropy gain by fibril formation. Since a recent estimate of the side-chain entropy loss by protein folding [55] is 2.8–4.2 JK^-1^mol^-1^ per residue at 300 K, the entropy loss by these 23 residues is 0.24–0.69 JK^-1^mol^-1^ per residue.

Using the minimum value of the side-chain entropy gain, 10.4 (= 13.6 − 3.2) JK^-1^mol^-1^ per residue, the net gain of the conformational entropy at 300 K is at least 2.9 (= 10.4–6.8–0.69) JK^-1^mol^-1^ per residue, which suggests that the fibril formation increases the conformational entropy. Because the hydration entropy also increases with fibril formation [54], the fibril formation is suggested to be entropically favorable.

An enthalpy change by fibril formation is estimated from isothermal titration calorimetry measurements [31] to be 32 kJmol^-1^ at 300 K (extrapolated from the values reported). The conformational entropy gain of 2.9 JK^-1^mol^-1^ per residue provides the TΔS_Conf_ value of 122 kJmol^-1^ at 300 K, which indicates that the main contribution to the free energy is the entropy term. This has an implication that once a potential barrier is overcome, fibril formation proceeds naturally. The rate-limiting step of fibril formation would then be the kinetics involved to overcome the potential barrier.

From the biophysical characterization of the dynamical behavior of αSyn described above, inference could be made concerning possible relevance of the dynamical behavior to the pathological aspects of the disease. Without any external triggers such as agitation, fibril formation of αSyn takes months. However, fibril formation is greatly accelerated by various factors, including external forces such as agitation and environmental conditions such as ionic strength, metal concentration, pH, temperature, and molecular crowding [57]. Moreover, the mutants of αSyn related to familial PD, such as A30P and A53T, have an increased propensity to form fibrils [58]. Such external triggers and environmental conditions appear to be factors that affect the potential barrier against the fibril formation. Considering the fact that sporadic PD is an age-related late-onset disease whereas the familial PD, in which the mutations above were identified, is an early-onset disease, the onset of the symptom of PD may depend critically on this kinetic step to overcome the potential barrier. Conversely, avoiding environmental risks that may trigger fibril formation could avoid the onset of (sporadic) PD. In this regard, the fact that exposure to heavy metals such as aluminum, copper, iron, lead, and manganese is a risk factor for PD may be an example because these heavy metals accelerate the rate of the fibril formation [59].

The local motion with larger amplitude in the fibril state than that in the monomeric state observed here is consistent with the results of concanavalin A amyloid fibrils obtained by the neutron scattering measurements on hydrated powder samples, which revealed an enhanced dynamics in the fibrils [60]. We also obtained similar results from the neutron scattering measurements on a model system for amyloid fibril formation, hen egg-white lysozyme in water-ethanol mixtures [36]. On the other hand, as shown, for example, by neutron scattering studies of actin [61,62], which polymerizes into filamentous polymers through a "normal" mechanism, ordered structures usually have lower flexibility. As pointed out in Ref. 60, the enhanced dynamics in amyloid fibrils contradicts this usually accepted paradigm. Since amyloid fibrils form in a number of proteins that are not related to each other structurally or functionally at all [63], this "abnormal" dynamics may well be relevant to the general mechanism of amyloid fibril formation.

## Supporting Information

S1 Fig Characterization of αSyn in the monomeric and fibril states. (a) Example of CD spectra of αSyn in the monomeric state and (b) in the fibril state. (c) Example of the number distribution of the hydrodynamic radius of αSyn in the monomeric state, obtained from DLS measurements. Data at the scattering angle of 90° at 15°C are shown. A solid line in black denotes the experimental distribution, and a broken line in blue denotes the Gaussian fit to the peak region of the experimental distribution. Assuming that this deviation arises from the oligomers, the ratio of the area defined by the Gaussian to the area defined by the experimental distribution can be regarded as a fraction of the monomers in the sample. This ratio was calculated to be 0.89, indicating that about 90% of the sample is in the monomeric state. (d) TEM image of αSyn in the fibril state.
(DOCX)

S2 Fig Summary of the examples of quasielastic neutron scattering spectra. (a) The QENS spectra, S(Q,ω), of αSyn in the monomeric state and (b) those in the fibril state, at Q = 1.225 Å^-1^, at each temperature measured, are summarized. Open squares are the experimental spectra, solid lines in black denote the total fits, solid lines in green and red denote the narrow and wide Lorentzian functions, corresponding to *L*_global_(Q,ω) and *L*_local_(Q,ω), respectively, thin solid lines in black show the background, and dashed lines in black show the resolution functions.
(DOCX)

S1 Text Details of data reduction of quasielastic neutron scattering spectra. (DOCX)

S2 Text Discussion of possible errors in quasielastic neutron scattering spectra. (DOCX)
