# Supplementary figures and images for: Dynamical Behavior of Human α-Synuclein Studied by Quasielastic Neutron Scattering
Source: PLoS One. 2016 Apr 20;11(4):e0151447. doi: 10.1371/journal.pone.0151447 (PMC4838215; doi:10.1371/journal.pone.0151447)

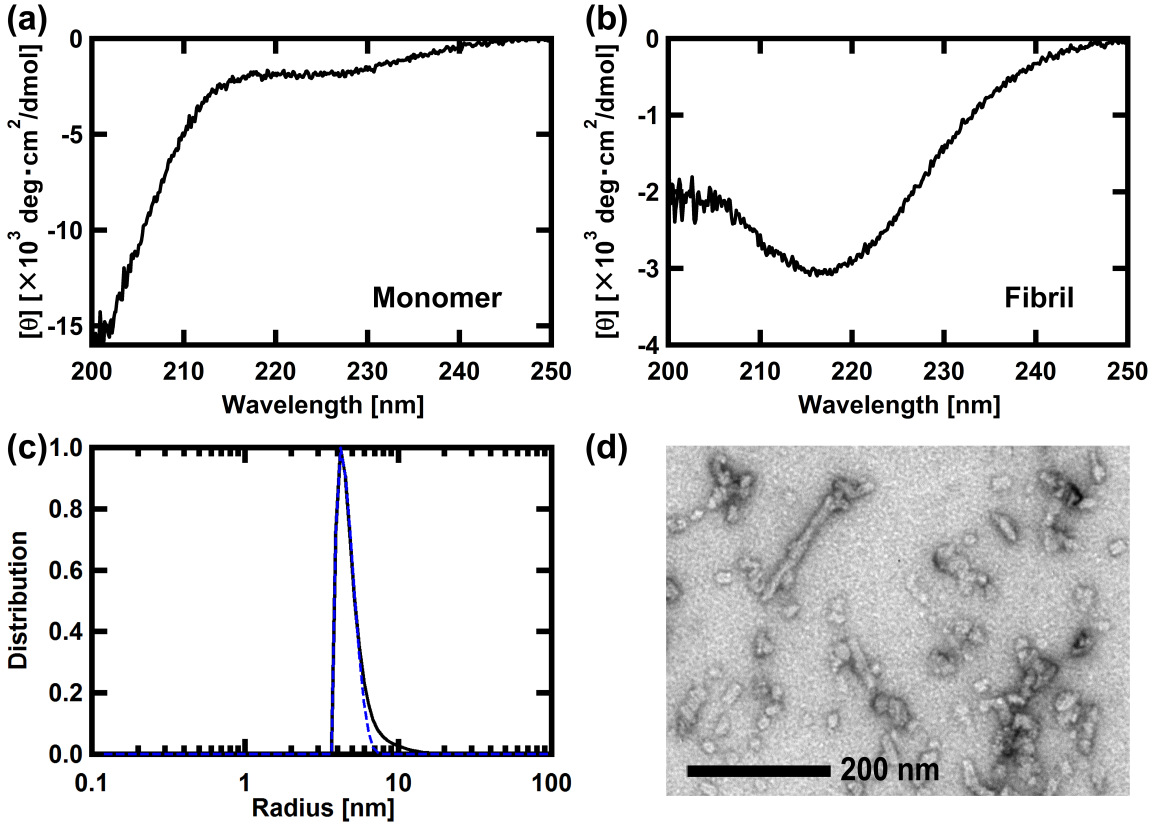


**S1 Fig. Characterization of αSyn in the monomeric and fibril states.**

Supplement: S1 Fig — (a) Example of CD spectra of αSyn in the monomeric state and (b) in the fibril state. (c) Example of the number distribution of the hydrodynamic radius of αSyn in the monomeric state, obtained from DLS measurements. Data at the scattering angle of 90° at 15°C are shown. A solid line in black denotes the experimental distribution, and a broken line in blue denotes the Gaussian fit to the peak region of the experimental distribution. Assuming that this deviation arises from the oligomers, the ratio of the area defined by the Gaussian to the area defined by the experimental distribution can be regarded as a fraction of the monomers in the sample. This ratio was calculated to be 0.89, indicating that about 90% of the sample is in the monomeric state. (d) TEM image of αSyn in the fibril state. (DOCX) [file pone.0151447.s001.docx]

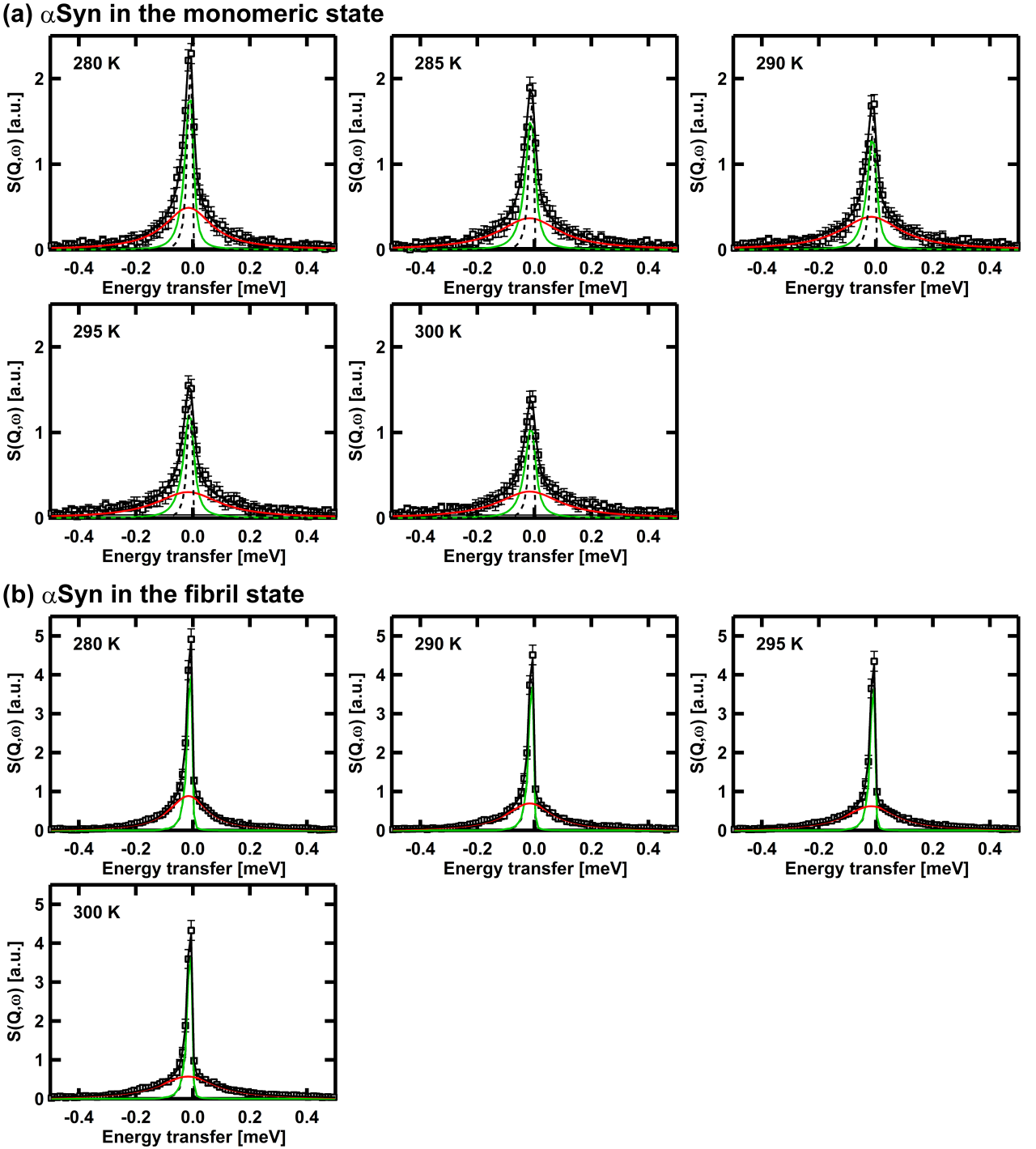


**S2 Fig. Summary of the examples of quasielastic neutron scattering spectra.**

Supplement: S2 Fig — (a) The QENS spectra, S(Q,ω), of αSyn in the monomeric state and (b) those in the fibril state, at Q = 1.225 Å-1, at each temperature measured, are summarized. Open squares are the experimental spectra, solid lines in black denote the total fits, solid lines in green and red denote the narrow and wide Lorentzian functions, corresponding to Lglobal(Q,ω) and Llocal(Q,ω), respectively, thin solid lines in black show the background, and dashed lines in black show the resolution functions. (DOCX) [file pone.0151447.s002.docx]
