# Supplementary material for: Dynamical Behavior of Human α-Synuclein Studied by Quasielastic Neutron Scattering
Source: PLoS One. 2016 Apr 20;11(4):e0151447. doi: 10.1371/journal.pone.0151447 (PMC4838215; doi:10.1371/journal.pone.0151447)
Supplement: S1 Text — (DOCX) [file pone.0151447.s003.docx]

**S1 Text. Details of data reduction of quasielastic neutron scattering spectra**

To extract the quasielastic neutron scattering (QENS) spectra arising from the internal dynamics of αSyn, the QENS spectra of the buffer were subtracted from those of the αSyn solution sample, based on the incoherent scattering cross-section of the protein and the buffer.

Based on the amino acid sequence of αSyn (UniProt identifier P37840), the chemical composition of αSyn is C_627_H_784_D_228_N_166_O_216_S_4_, assuming that all the exchangeable hydrogen are replaced with deuterium, and without taking account of protonation and dissociation. The incoherent scattering cross-section Σ^inc^ of αSyn can be calculated from this chemical composition and the incoherent scattering cross-section σ^inc^_atom_ of the atoms in the protein (σ^inc^_H_ = 79.9 barn or 10^-24^ cm^2^, σ^inc^_D_ = 2.04 barn, σ^inc^_C_ = 0.001 barn, σ^inc^_N_ = 0.49 barn, σ^inc^_O_ = 0.0 barn, and σ^inc^_S_ = 0.007 barn, taken from Table 2.2 in Ref. 37 in the main text), using the equation,

Σ^inc^ = Σ mol_atom_*N_A_*σ^i^_atom_ ,

^atom^

where mol_atom_ is the molar number of the atoms, and *N_A_* is the Avogadro's number. Summation was done over all atoms in the protein. For the monomeric state of αSyn, where the concentration of αSyn was 9.5 mg/ml, Σ^inc^ for αSyn in unit volume (1 cm^3^) is calculated to be 0.0246 cm^-1^. Assuming that the partial specific volume of αSyn is 0.73, the volume fraction of the solvent in the unit volume is calculated to be 1− 0.73×9.5×10^-3^ ≈ 0.993. Assuming, then, that the density of D_2_O is 1.11 g/cm^3^ and using this volume fraction, the incoherent scattering cross-section of D_2_O in the solvent is calculated to be 0.135 cm^-1^. Note that although the solvent contains salts (10 mM Na_2_DPO_4_, 1.76 mM KD_2_PO_4_, 137 mM NaCl, and 2.7 mM KCl), the contribution of these salts to the incoherent scattering cross-section is negligible compared with that of D_2_O because the incoherent scattering cross-section of each salt is less than 10^-3^ cm^-1^ (Na_2_DPO_4_: 4.8×10^-4^ cm^-1^, KD_2_PO_4_: 1.6×10^-4^ cm^-1^, NaCl: 5.4×10^-4^ cm^-1^, and KCl: 8.6×10^-6^ cm^-1^). The fraction of the contribution of the solvent to the spectra of αSyn in the monomeric state is thus 0.846 (= 0.135/(0.135+0.0246)). This value is used as a scaling factor to subtract the buffer spectra from the sample spectra.

A similar calculation for the fibril state of αSyn, in which the concentration of αSyn is 46 mg/ml, reveals that the incoherent scattering cross-section are 0.119 cm^-1^ and 0.132 cm^-1^ for αSyn and D_2_O, respectively. The scaling factor for the subtraction of the buffer spectra is thus 0.526 (= 0.132/(0.132+0.119)).

The transmission T can be calculated from the equation, T = exp(-Σd), where Σ is the total scattering cross-section of the sample, and d is the thickness of the sample. Σ is the sum of the incoherent scattering cross-section (Σ^inc^), the coherent scattering cross-section (Σ^coh^), and the absorption cross-section (Σ^abs^) of the sample. Σ^inc^ for αSyn and D_2_O were calculated above. Σ^coh^ and Σ^abs^ for αSyn and D_2_O can be calculated similarly using, instead of the values of σ^inc^_atom_, the values of the coherent scattering cross-section σ^coh^_atom_ of the atoms (σ^coh^_H_ = 1.759 barn, σ^coh^_D_ = 5.597 barn, σ^coh^_C_ = 5.554 barn, σ^coh^_N_ = 11.01 barn, σ^coh^_O_ = 4.235 barn, and σ^coh^_S_ = 1.019 barn) and those of the absorption cross-section σ^abs^_atom_ of the atoms (σ^abs^_H_ = 0.333 barn, σ^abs^_D_ = 0.0005 barn, σ^abs^_C_ = 1.90 barn, σ^abs^_N_ = 1.90 barn, σ^abs^_O_ = 0.0002 barn, and σ^abs^_S_ = 0.53 barn; the values of σ^coh^_atom_ and σ^abs^_atom_ are taken from Table 2.2 in Ref. 19 in the main text). Σ (= Σ^inc^_αSyn_ + Σ^inc^_D2O_ + Σ^coh^_αSyn_ + Σ^coh^_D2O_ + Σ^abs^_αSyn_ + Σ^abs^_D2O_) are calculated to be 0.765 cm^-1^ and 0.678 cm^-1^ for the fibril-state sample and the monomeric-state sample, respectively. Using the sample thickness of 0.5 mm for the fibril-state sample and 1.0 mm for the monomeric-state sample, the transmission is calculated to be 0.962 and 0.934 for the fibril-state sample and the monomeric-state sample, respectively. The effect of multiple scattering should be negligible for samples with such high transmission (see S2 Text).
