# Supplementary material for: Dynamical Behavior of Human α-Synuclein Studied by Quasielastic Neutron Scattering
Source: PLoS One. 2016 Apr 20;11(4):e0151447. doi: 10.1371/journal.pone.0151447 (PMC4838215; doi:10.1371/journal.pone.0151447)
Supplement: S2 Text — (DOCX) [file pone.0151447.s004.docx]

**S2 Text. Discussion of possible errors in quasielastic neutron scattering spectra**

Although the QENS spectra arising from αSyn are well above the noise level as shown in Fig 1 in the main text, various systematic errors may distort the spectra. We discuss here the various possible errors, which may be due to (1) the contribution of coherent scattering, (2) the contribution of the spectra of the empty cell, (3) the effect of the shape of the sample cell, (4) multiple scattering, and (5) the scaling of the spectra of the buffer for subtraction.

*Possibility (1)*:

As calculated above, the incoherent scattering cross-section and the coherent scattering cross-section of αSyn in the monomeric state are 0.0246 cm^-1^ and 0.00346 cm^-1^, respectively, and those in the fibril state are 0.119 cm^-1^ and 0.0168 cm^-1^, respectively. About 10% of the total scattering thus arises from the coherent scattering. The intensity of the coherent scattering, however, falls rapidly with increasing Q because the structure factor arising from the interferences between different atoms contributes to coherent scattering [1]. The contribution of coherent scattering becomes negligible for Q>~0.4 [2]. Our analysis involves the region where coherent scattering is negligible. Thus, coherent scattering did not introduce appreciable error.

*Possibility (2)*:

We examined this possibility by carrying out subtraction of the spectra of the empty cell with various scaling factors. We carried out the subtraction with scaling factors in the range of ±10% around the transmission of the samples (but in the range below 1.0 because the transmission larger than 1.0 is physically impossible). The spectra obtained with different scaling factors are essentially indistinguishable because the contribution of the empty cell is largely canceled by subtracting the buffer spectra from the sample spectra, both of which contain equal contributions of the spectra of the empty cell. Furthermore, we found that the spectra of the cells of the same geometry, made of the same material, are also practically indistinguishable. Use of distinct cells for the samples and the buffers thus has negligible effects. Thus, errors due to the spectra of the empty cell were negligible.

*Possibility (3)*:

Double cylindrical cells were used for the measurements of αSyn in the monomeric state while flat cells were used for the measurements of αSyn in the fibril state. The data obtained using the cells with different shapes were reduced separately. The spectra were normalized by those of vanadium standard obtained from vanadium plates with the same shape as the sample cells (a cylinder for the double cylindrical cell and a flat plate for the flat cell). The effects of the shape of the vanadium standard should be reflected in the spectra of the vanadium standard. Normalization by these vanadium spectra thus eliminates the effects of the shape. Possible differences due to the different shapes of the sample cells were eliminated by this normalization.

*Possibility (4)*:

Common practice of the QENS measurements is that if the transmission of a sample is greater than 0.90, effects of multiple scattering can be neglected [3]. Many studies follow this "rule of thumb" (for example, Refs 12, 17, 18, 22, 24, 26, 35, 38, 46, 47, and 60 in the main text explicitly stated that this rule applies.) As shown above, the transmission of the samples used here was well above 0.9. Thus, the effects of multiple scattering are neglected.

Furthermore, the effects of multiple scattering are shown to be prominent in the low-Q region [4]. Observation of the slight over-broadening of the QENS spectra at the region Q < 0.5 Å^-1^ for the samples with the transmission lower than 0.90 may indeed be due to the effects of multiple scattering [5]. Since the spectra in this study were analyzed for the range Q > 0.45 Å^-1^, the few points in the lowest Q could be affected by multiple scattering. However, the parameters do not show appreciable deviations from the general tendency to the Q-dependences (see, Figs 2, 3, and 4 in the main text), and omitting these points from the fits (where applicable) did not change the results obtained. This is an indication that the effects of multiple scattering are indeed negligible.

*Possibility (5)*:

Various sources of error, such as errors in the protein concentration and partial specific volume, can introduce error into the scaling factor for subtracting the buffer spectra from the sample spectra. Error in this scaling factor could thus be most significant. We checked this possibility by subtracting the buffer spectra with various scaling factors in the range of ±10% around the value determined as described above from the scattering cross-section and with the same analysis applied to these spectra as described in the manuscript. Analysis shows that the obtained parameters (the diffusion coefficient *D_T_* of the global motion, the activated energy *E*_a_ estimated from the residence time of the local motion, and the fraction *p* of the frozen atoms, and the radius *a* of a confined sphere estimated from the EISF curves) were within the errors of the values obtained from the "original" spectra, which were obtained as described in Materials and Methods and S1 text, except for *p* in the monomeric state. Regarding *p*, although the range of *p* in the monomeric state was somewhat wider than the range of error in the original values, the result that *p* in the monomeric and fibril states had similar values did not change. Note also that regardless of the scaling factors, the result for *a* for the monomeric and fibril states remain different.

We thus conclude that these possible systematic errors do not affect the results of our analysis. The differences in the spectra and the parameters obtained from analyzing the spectra indeed reflect the differences in the dynamics between αSyn in the monomeric and fibril states.

**References**

1. Gasper AM, Busch S, Appavou MS, Haeussler W, Georgii R, Su Y et al. Using polarization analysis to separate the coherent and incoherent scattering from protein samples. Biochem. Biophys. Acta. 2010; 2804: 76-82.
2. Fujiwara S, Yamada T, Matsuo T, Takahashi N, Kamazawa K, Kawakita Y et al. Internal dynamics of a protein that forms the amyloid fibrils observed by neutron scattering. J Phys Soc Jpn. 2013; 82: SA019.
3. Fitter J. Conformational dynamics measured with proteins in solution. In Neutron Scattering in Biology Techniques and Applications. J. Fitter, T. Gutberlet, and J. Katsaras, editors. Springer Berlin Heidelberg. 2006; 399-418.
4. Wuttke J. Multiple-scattering effects on smooth neutron-scattering spectra. Phys. Rev. E 2000; 62: 6531
5. Ishai PB, Mamontov E, Nickels JD, Sokolov AP. Influence of ions on water diffusion – a neutron scattering study. J. Phys. Chem. B 2013; 11: 7724-7728
